# Vultures acquire information on carcass location from scavenging eagles

**DOI:** 10.1098/rspb.2014.1072

**Published:** 2014-10-22

**Authors:** Adam Kane, Andrew L. Jackson, Darcy L. Ogada, Ara Monadjem, Luke McNally

**Affiliations:** 1 Department of Zoology, School of Natural Sciences, Trinity College Dublin, Dublin 2, Republic of Ireland; 2 Centre for Biodiversity Research, Trinity College Dublin, Dublin 2, Republic of Ireland; 3 The Peregrine Fund, 5668 West Flying Hawk Lane, Boise, ID 83709, USA; 4 National Museums of Kenya, Ornithology Section, PO Box 40658, Nairobi 00100, Kenya; 5 All Out Africa Research Unit, Department of Biological Sciences, University of Swaziland, Private Bag 4, Kwaluseni, Swaziland; 6 Centre for Immunity, Infection and Evolution, School of Biological Sciences, University of Edinburgh, Edinburgh EH9 3JT, UK; 7 Institute of Evolutionary Biology, School of Biological Sciences, University of Edinburgh, Edinburgh EH9 3JT, UK

**Keywords:** inter-guild interactions, producer–scrounger game, scavengers, social information, vultures

## Abstract

Vultures are recognized as the scroungers of the natural world, owing to their ecological role as obligate scavengers. While it is well known that vultures use intraspecific social information as they forage, the possibility of inter-guild social information transfer and the resulting multi-species social dilemmas has not been explored. Here, we use data on arrival times at carcasses to show that such social information transfer occurs, with raptors acting as producers of information and vultures acting as scroungers of information. We develop a game-theoretic model to show that competitive asymmetry, whereby vultures dominate raptors at carcasses, predicts this evolutionary outcome. We support this theoretical prediction using empirical data from competitive interactions at carcasses. Finally, we use an individual-based model to show that these producer–scrounger dynamics lead to vultures being vulnerable to declines in raptor populations. Our results show that social information transfer can lead to important non-trophic interactions among species and highlight important potential links among social evolution, community ecology and conservation biology. With vulture populations suffering global declines, our study underscores the importance of ecosystem-based management for these endangered keystone species.

## 1. Introduction

Animals base their decisions on both personal and public information [1–4]. This is applicable to every facet of an animal's life, be it feeding, movement, mating, etc., with high fidelity information allowing an individual to make decisions conducive to its survival [1,5,6]. Public information can be separated into that which is gained from intraspecifics and that from interspecifics [1]. Intraspecific information transfer is essential for basic behavioural functions such as sexual reproduction or cooperative hunting [7]. But species overlap in the resources they use [8,9] and the environments they inhabit [8,9], which gives the possibility of inter-guild information transfer [10–12].

Consider the social *Gyps* vultures, a group that is known to forage collectively for carrion. In flight, they appear to keep in visual contact with conspecifics [13]. Once one vulture discovers and descends to a carcass, the information is conveyed to others in the area; this activity can create a local enhancement effect [14]. But such social behaviour renders vultures' foraging efficiency susceptible to population declines; with every individual lost, the network is less effective at detecting carrion [14].

Although vultures are the most well-known group of avian scavengers, there are a number of other species within the family Accipitridae, such as eagles (hereafter raptors), that take carrion as a significant proportion of their diet [15]. Coexistence among all of these species is possible by both temporal and resource niche partitioning [15,16]. But with any shared resource, direct interactions between them will result, and the possibility of social information transfer among species emerges. A distinct pattern of arrival of avian scavengers to carrion has been highlighted before [17–19]. Indeed, the African white-backed vulture has been noted in using many other scavengers as a means of local enhancement while foraging [9]. Yet, these heterospecific interactions and their potential for information transfer have not been explored in any detail [17]. Given the current extreme declines in vulture populations [20,21] and their key role in many ecosystems as biomass recyclers [22,23], understanding vulture foraging ecology is also of applied relevance. Here, we provide evidence for producer–scrounger dynamics among scavenging vulture and raptor species by testing the hypothesis that vultures scrounge information from raptors and explore its evolutionary underpinnings using a game-theoretic model. We conclude by outlining the consequences of this system's properties for vulture conservation.

## 2. Test for producer–scrounger dynamics

To test for the occurrence of producer–scrounger dynamics between vultures and raptors, we observed arrival times of avian scavengers at a number of carcasses placed out (see the electronic supplementary material). Our observations were made on 46 videos recorded in the Mpala Research Centre in the Laikipia District of Kenya which had scavenging avifauna (a subset of the videos used in [22]). We focused on the closely related and morphologically similar *Gyps* vultures, the African white-backed vulture (*Gyps africanus*) and the Rüppell's vulture (*Gyps rueppellii*), as well as the congeneric tawny (*Aquila rapax*) and steppe eagles (*Aquila nipalensis*). These four species were by far the most abundant in the recordings (more than 95%) and formed our vulture and raptor groups, respectively [22].

**Figure 1.**
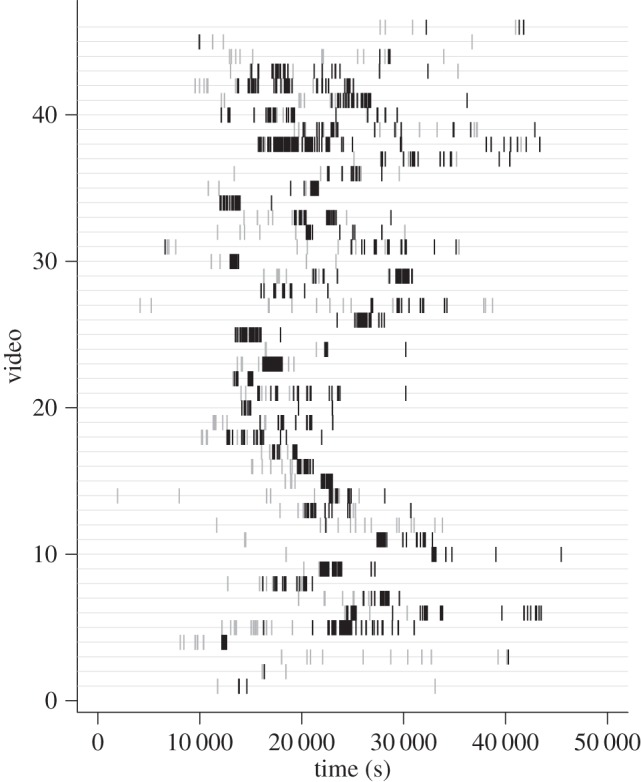
Recorded arrival times of individual vultures and raptors at carrion across 46 videos. The grey lines are the raptors and the black are the vultures.

For each video, we noted the arrival time and species of every animal. Initially, we compared the probability of producing information on carcass location by looking at which of the two groups, *Gyps* vultures or raptors, landed at the carcass first. A binomial test on the 46 videos showed that the first bird to land at a carcass was significantly more likely to be a raptor than a vulture (binomial test, 38 successes, 46 trials, expected probability = 0.5, results in observed probability = 0.83, 95% CI 0.69–0.92, *p*-value < 0.001; figure 1).

**Figure 2.**
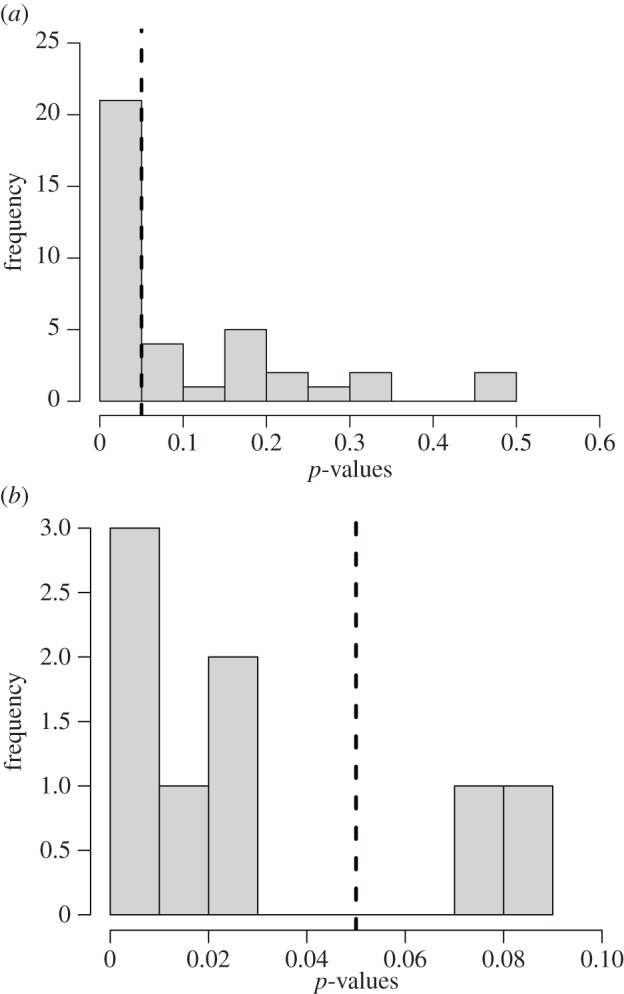
Histograms of *p*-values showing the number of videos where it was significantly probable that (*a*) the vultures were following the raptors and (*b*) the raptors were following the vultures. The vertical lines show the level of significance at *p* = 0.05.

We used randomization tests to test whether the birds were following each other rather than simply arriving independently but with different timing to the carcasses. Where a raptor landed first, we generated a null distribution of arrival times for the first scrounger (a *Gyps* vulture) over the length of each recording by randomizing the arrival times of the birds. From this distribution, we assessed whether the first scrounger followed more closely than expected under an assumption of independent foraging. Then, in order to make a population-level inference across permutation tests, we used a binomial test where the expected probability of observing a significant result by chance is 0.05 (as per the definition of a *p*-value, where at an *α* of 0.05, we would expect 5% of test results to be significant according to the null model). Vultures were found to follow raptors more closely than expected by chance (i.e. with *p* < 0.05) in 20 of the 38 videos (figure 2*a*), which is significantly more cases of vultures following raptors than expected (binomial test with 20 successes, 38 trials, expected probability = 0.05 results in an observed probability = 0.53, 95% CI 0.36–0.69, *p*-value < 0.001).

Similarly, on six of the eight occasions when vultures landed at the carcass first, raptors followed more closely than expected by chance (figure 2*b*), which is significantly more cases than expected (binomial test with six successes, eight trials, expected probability = 0.05 results in an observed probability = 0.75, 95% CI 0.35–0.97, *p*-value < 0.001).

## 3. Producer–scrounger model

While our analyses indicate that both raptors and vultures follow each other to carcasses, the higher frequency of raptors being the first to land at a carcass leads to raptors acting predominantly in a producing role. This can manifest by raptors providing information on the location of the resource or by engaging in carcass opening whereby the raptor uses its relatively stronger bill to get through an ungulate hide [18], with vultures acting predominantly as scroungers. This result raises the question of how did these divergent roles evolve? We hypothesized that competitive ability may have a strong effect on the strategy typically adopted in each species. If individuals of one species can competitively dominate those of another, this may favour scrounging by the dominant species: producers may gain an exclusive share of the resource by arriving first at the carcass (a ‘finder's fee’), but dominant scroungers may be able to effectively monopolize the remainder of the resource once they arrive, while also gaining information available publically on the locations of carcasses.

We used the game-theoretic framework of the producer–scrounger game to test the evolutionary feasibility of this prediction. In a producer–scrounger game, an animal needs to either invest in producing some resource (typically information) or exploit the investment from another individual [24]. We consider a scenario where vultures and raptors forage in the same area. We assume that time spent feeding at a carcass is small relative to search time [15,25]. Individual vultures and raptors can assume one of two strategies: producer or scrounger. Producers find carcasses at a rate proportional to the carcass density, while all scroungers in a group will follow producers to the carcasses they find, but do not find carcasses for themselves (probabilistic following by scroungers cannot qualitatively affect our results). While, in reality, vultures and raptors will likely use mixed strategies, this simple scenario allows us to abstract the essential elements of the evolutionary dynamics in a simple framework.

From our assumptions, we can write the numbers of vultures and raptors at a carcass found by a vulture as *m_v_*_,v_ = 1 + *v*_s_ and *m_v_*_,r_ = *r*_s_, respectively, where *v*_s_ and *r*_s_ are the numbers of vultures and raptors that are scroungers. Similarly, the numbers of vultures and raptors at a carcass found by a raptor are *m_r_*_,v_ = *v*_s_ and *m_r_*_,r_ = 1 + *r*_s_, respectively. We assume that new carcasses arrive in the area and decay at fixed rates (both set at 1) and are consumed almost instantaneously when found. Assuming that carcass dynamics occur on a faster timescale than the population dynamics of vultures and raptors, the steady-state density of carcasses is then given as *d* = 1/(1 + *r*_p_ + *v*_p_), where *v*_p_ and *r*_p_ are the numbers of vultures and raptors that are producers. The rates of food consumption for producing and scrounging vultures are3.1
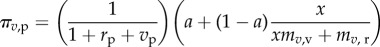
and3.2
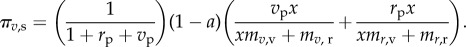
Similarly, the rates of food consumption for producing and scrounging raptors are3.3
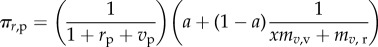
and3.4
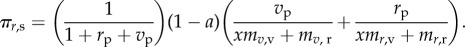
Here, *a* is the proportion of a carcass that is monopolized by the individual that finds it (the ‘finder's fee’), and *x* is the competitive ability of vultures compared with that of raptors, specifically the number of raptors that a vulture is equivalent to in terms of competitive ability. The proportion of the carcass remaining after the finder's fee (1 – *a*) is shared among all birds at the carcass proportionally to their relative competitive ability. For example, at a carcass found by a raptor, a scrounging vulture's share would be *x*/(*xm_r_*_,v_ + *m_r_*_,r_). The vulture is competitively equivalent to *x* raptors so gets a positive weighting of *x* in the numerator. As other vultures will have a similar competitive ability, the number of vultures at the carcass (*m_r_*_,v_) is also weighted by *x*. This leads to each bird receiving a share proportional to its competitive ability relative to the other birds present at the carcass. The probability that a vulture wins a one-on-one interaction with a raptor is then defined as *x*/(1 + *x*). This process could then be seen as a series of competitive interactions over small proportions of the carcass, leading to birds on average receiving a share proportional to their relative competitive ability.

While the equilibrium number of carcasses (1/(1 + *r*_p_ + *v*_p_)) available declines with the density of producers of both species as more carcasses are found and consumed, the food acquisition rate of scroungers is also positively weighted by producer densities as they are able to follow individuals to carcasses more frequently. This means that, while producers are only affected negatively by other producers (owing to reduction in carcass densities), scroungers are affected both positively (owing to their increasing rate of following to carcasses) and negatively (owing to reduction in carcass density) by producer density.

We write the dynamics [26] of producers and scroungers in the vulture and raptor populations as3.5
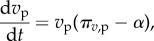
3.6
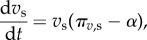
3.7
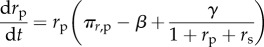
3.8
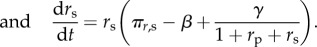
Here, *α* and *β* are the mortality rates for vultures and raptors, respectively. The additional term *γ*/(1 + *r*_p_ + *r*_s_) captures additional food intake by raptors owing to their additional source of energy through predation. Here, we assume that some prey enters the area at rate *γ*, dies at a fixed rate of 1 and is found by raptors at rate 1 and then instantaneously consumed. Again we assume that the dynamics of the prey population happen on a faster timescale than the raptor population dynamics so that the steady-state density of prey can be used. Varying the parameter *γ* then allows us to vary raptor's relative reliance on carcasses as a food source.

**Figure 3.**
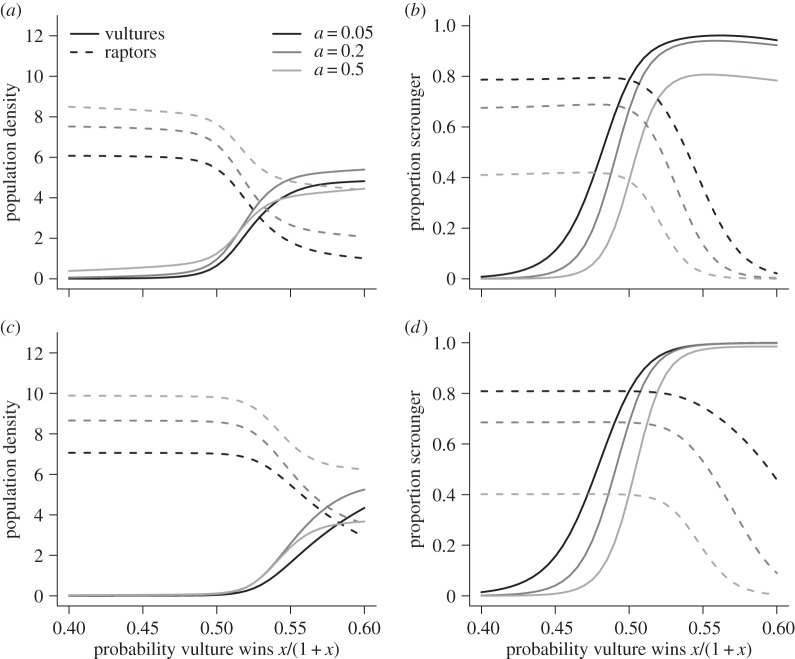
Game theory results. (*a*,*c*) Population densities for each species (vultures, solid lines; raptors, dashed lines), and (*b*,*d*) frequencies of scroungers in both the vulture and raptor populations. (*a*,*b*) Raptors rely strongly on carcasses (*γ* = 0.05); (*c*,*d*) they rely more weakly on carcasses (*γ* = 0.15). Line shades indicate different values for the finder's fee (black, *a* = 0.05; dark grey, *a* = 0.2; light grey, *a* = 0.5). The *x*-axis is the probability that a vulture wins a one-on-one interaction, *x*/(1 + *x*). Mortality rates are *α* = 0.1 and *β* = 0.1 for all panels.

Unfortunately, no analytical solutions are available for our model, so we examine the evolutionary dynamics of the producer–scrounger interaction using numerical evaluation of steady-state of equations (3.5)–(3.8). The results of the model displayed in figure 3 show the impact of competitive ability, finder's advantage and the availability of prey items to raptors. Note that in figure 3*a*,*c*, there is a transition from high raptor population densities to high vulture densities as vultures become more dominant over raptors (the switch occurring when the probability a vulture wins is greater than 0.5). The availability of extra food from predation in figure 3*c* allows the raptors to persist at higher population densities, suppressing the increasing vulture numbers relative to figure 3*a*. The effect of increasing relative competitive ability is also realized in driving up the *proportion* of birds scrounging. The outcome of varying the size of the finder's fee is evident as we can see a lower proportion of scroungers when the amount of food consumed by the producer is high. A competitively dominant species gains a larger share of the resource. It follows that any finder's fee is of less value to them than it is to the competitively inferior species. Thus the competitively dominant species is more likely to forego a finder's fee in order to benefit from the increased rate of information acquisition that can be facilitated by scrounging.

## 4. Test of competitive ability

The results of our model demonstrate the potential importance of competitive asymmetry in the evolutionary outcome of inter-guild producer–scrounger dynamics. To test our model prediction of competitive dominance by vultures, we analysed competitive interactions between *Gyps* and raptor species at carcasses from our videos. We followed Bamford *et al.* [27] in our analysis of agonistic interactions between the birds. In each case of aggression, we noted the initiator and the winner. The loser was defined as a bird spatially displaced by the direct action of another individual.

**Figure 4.**
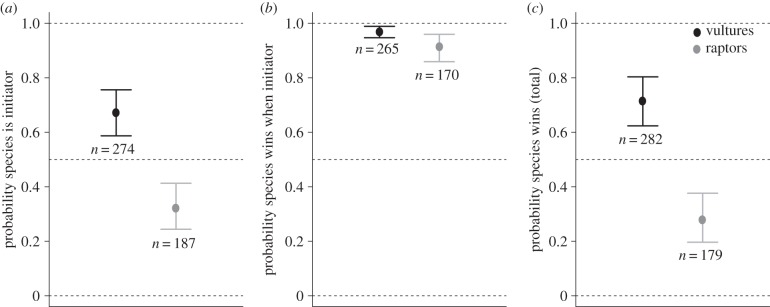
The results of the competitive interactions show (*a*) the probability that a species was an initiator, (*b*) the probability that an initiator wins a contest, and (*c*) the overall competitive ability. Given are the means with 95% CIs.

There were 461 interactions in total. We used a binomial generalized linear mixed model with video as a random effect to test the significance of the interactions (figure 4). In support of our theoretical predictions, we found vultures are more likely to be the initiator of an aggressive interaction (*n* = 274 versus 187, figure 4*a*, *β* = 0.7414, s.e. = 0.1987, *p* < 0.001, probability = 0.68, 95% CI 0.59–0.76); vultures are more likely to win when they initiate the contest (*n* = 265/274, figure 4*b*, *β* = 3.6942, s.e. = 0.4134, *p* < 0.001, probability = 0.98, 95% CI 0.95–0.99) and raptors are more likely to win when they initiate the contest (*n* = 170/187, *β* = 2.4893, s.e. = 0.3473, *p* < 0.001, probability = 0.92, 95% CI 0.86–0.96). The probability that a vulture wins when it initiates a contest is also significantly greater than the probability that a raptor wins when it is the initiator (*β* = 1.2049, s.e. = 0.4685, *p* = 0.0101). Finally, vultures are more likely to win overall (*n* = 282 versus 179, figure 4*c*, *β* = 0.9567, s.e. = 0.2303, *p* < 0.001, probability = 0.72, 95% CI 0.62–0.80).

## 5. Effects of raptor density on vulture foraging efficiency

The producer–scrounger dynamics that we have illustrated suggest a possible ecological interaction whereby vultures are using raptors to locate carcasses. This would imply that vultures may be vulnerable to declines in raptor populations as their ability to locate food will also decline. To examine this possibility, we created an individual-based model (IBM) in the program NetLogo [28] to explore the effect of raptors on the foraging efficiency of the vultures. Our model is a modified version of Jackson *et al*. [14] and Dermody *et al*. [29], both of which examined vulture foraging behaviour. The main difference is that we include raptors alongside vultures.

Our video analysis suggests raptors can find carcasses before vultures. The question is what is it about their biology that allows them to achieve this? A recent study found that lappet-faced vultures can discover carrion before white-backed vultures despite their smaller population size [30]. We incorporate the changes they deemed likely to impact differential search efficiencies applicable to raptors, namely, visual acuity [31], flying height, roost departure time and dispersion of the birds at the start of the foraging day owing to different roost arrangements (see the electronic supplementary material).

At the beginning of the IBM, the raptors were randomly allocated in the simulation space that corresponds to a square of 100 × 100 km with periodic boundary conditions so that a bird that flies off the edge of the square will reappear on the opposite side. The vultures are located in a single patch which represents their roost. The raptors forage for 7 h and the vultures 5 h [30]. The vultures change direction by 45° once every 8 min, which is based on the time they spend in thermals [32]; as raptors are less dependent on thermals, they change direction at double this rate. Both have a constant speed as they attempt to find a single randomly located carcass [29]. Both vultures and raptors can find the carrion by themselves. We varied the relative detection distances between the groups such that they are equal; and then that raptors are two, three and four times better. For each of these, we varied the number of raptors from 1 to 10 relative to the 90 vultures present in the simulation (see the electronic supplementary material, table S2).

In the model, vultures can detect carrion at 1 km and can detect other scavengers on a carcass at 4 km [29], the increase in the latter owing to local enhancement [14]. In the version of the model whereby raptors have double the detection distance they can detect carrion at 2 km, which gives a fourfold increase in search area. When a vulture discovers a carcass, it ‘feeds’ on it, with the model calculating the average amount of food eaten by the vultures at the end of the simulated foraging session. Each simulation was replicated 200 times. We square-root transformed our data and analysed it using generalized linear models (GLMs).

**Figure 5.**
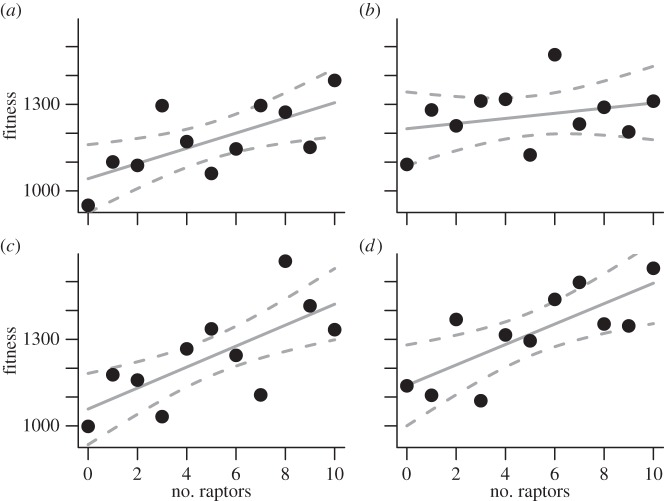
Mean vulture food intake (arbitrary units) as a function of raptor number for four scenarios of differing relative detection distance. (*a*) Equal distance (GLM, *β* = 0.3214, s.e. = 0.1232, *p* = 0.00916), (*b*) twice distance (GLM, *β* = 0.3269, s.e. = 0.123, *p* = 0.00794), (*c*) triple distance (GLM, *β* = 0.5047, s.e. = 0.1198, *p* < 0.001) and (*d*) quadruple distance (GLM, *β* = 0.6052, s.e. = 0.1254, *p* < 0.001).

Our simulation results show a significant increase in vulture foraging efficiency with raptor density (figure 5), indicating that declines in raptor numbers may lead to declines in vulture populations because of a reduced ability to find or open carcasses.

## 6. Discussion

Our results suggest that there is a producer–scrounger game occurring between *Gyps* vultures and scavenging raptors, with the competitive dominance of vultures favouring a scrounging strategy on their part.

The biology of the two groups further lends itself to the evolution of producer–scrounger dynamics. *Gyps* vultures are dependent on thermals to fly [33]. Flapping flight is far more energetically expensive than thermal soaring [34] and would prevent vultures from exploring a sufficient area to be effective scavengers [33]. Although raptors do exploit thermals as well, their relatively small size allows them to use the weaker early morning thermals compared with the larger vultures [35]. Thus they are likely to encounter carrion before the vultures. Kendall [18] found that, for their abundance, tawny eagles were more likely to discover a carcass than African white-backed vultures, and Ruppell's vultures were never the first to arrive at a carcass, which is consistent with producer–scrounger dynamics. She also reported several cases whereby the African white-backed vultures would not feed at a carcass until a tawny eagle began to eat. As mentioned earlier, this may be an instance of carcass opening [18]. The *Gyps* vultures can then dominate the raptor and feed on the previously inaccessible flesh. This would certainly qualify as a producer–scrounger system.

The proposed dynamics are not the result of an abundance of raptors happening upon carcasses more often than the vultures because raptors occur at much lower densities. In the Masai Mara, for instance, *Gyps* species were recorded at an average density of 85.4 species per 100 km compared with 7.4 for tawny eagles [36].

In sum, we show that foraging behaviour in *Gyps* vultures is more complex than previously thought. Social information transfer flows within and among the vulture and raptor species. In conservation terms, the resultant non-trophic interactions [37] mean that we should shift our focus to ecosystem-based management [38,39] instead of centring our attention on one species at a time. As our IBM shows, scrounging vultures will fare poorly with a decline in producing raptors. With raptor populations on the decline [40], this effect may soon be realized. More generally, we should explore other incidences of socially acquired information transfer between species: inadvertent as it often is, this will be no easy task.

## Supplementary Material

Supplementary Information

## Supplementary Material

Supplementary Data - competitive interactions

## Data accessibility

Some of the data used in the analyses are available in the electronic supplementary material.

## References

[1] Dall SRX Giraldeau LA Olsson O McNamara JM Stephens DW 2005 Information and its use by animals in evolutionary ecology. Trends Ecol. Evol. 20, 187–193. (10.1016/j.tree.2005.01.010) 16701367

[2] Schmidt KA Dall SR Van Gils JA 2010 The ecology of information: an overview on the ecological significance of making informed decisions. Oikos 119, 304–316. (10.1111/j.1600-0706.2009.17573.x)

[3] Sumpter D Buhl J Biro D Couzin I 2008 Information transfer in moving animal groups. Theor. Biosci. 127, 177–186. (10.1007/s12064-008-0040-1) 18458976

[4] Couzin ID 2009 Collective cognition in animal groups. Trends Cogn. Sci. 13, 36–43. (10.1016/j.tics.2008.10.002) 19058992

[5] McNamara JM Dall SR 2010 Information is a fitness enhancing resource. Oikos 119, 231–236. (10.1111/j.1600-0706.2009.17509.x)

[6] Danchin É Giraldeau L-A Valone TJ Wagner RH 2004 Public information: from nosy neighbors to cultural evolution. Science 305, 487–491. (10.1126/science.1098254) 15273386

[7] Handegard NO Boswell KM Ioannou CC Leblanc SP Tjøstheim DB Couzin ID 2012 The dynamics of coordinated group hunting and collective information transfer among schooling prey. Curr. Biol. 22, 1213–1217. (10.1016/j.cub.2012.04.050) 22683262

[8] Fedriani JM Fuller TK Sauvajot RM York EC 2000 Competition and intraguild predation among three sympatric carnivores. Oecologia 125, 258–270. (10.1007/s004420000448) 24595837

[9] Kruuk H 1967 Competition for food between vultures in East Africa. Ardea 55, 171–193.

[10] Seppänen J-T Forsman JT Mönkkönen M Thomson RL 2007 Social information use is a process across time, space, and ecology, reaching heterospecifics. Ecology 88, 1622–1633. (10.1890/06-1757.1) 17645008

[11] Seppänen J-T Forsman JT 2007 Interspecific social learning: novel preference can be acquired from a competing species. Curr. Biol. 17, 1248–1252. (10.1016/j.cub.2007.06.034) 17614285

[12] Forsman JT Hjernquist MB Gustafsson L 2009 Experimental evidence for the use of density based interspecific social information in forest birds. Ecography 32, 539–545. (10.1111/j.1600-0587.2008.05635.x)

[13] Houston D 1974 Food searching in griffon vultures. Afr. J. Ecol. 12, 63–77. (10.1111/j.1365-2028.1974.tb00107.x)

[14] Jackson AL Ruxton GD Houston DC 2008 The effect of social facilitation on foraging success in vultures: a modelling study. Biol. Lett. 4, 311–313. (10.1098/rsbl.2008.0038) 18364309 PMC2610049

[15] Mundy PJ Butchart D Ledger J Piper S 1992 The vultures of Africa. London, UK: Academic Press.

[16] Houston D 1975 Ecological isolation of African scavenging birds. Ardea 63, 55–64.

[17] Mundy PJ 1982 *The comparative biology of southern African vultures*. Johannesburg, South Africa: Vulture Study Group.

[18] Kendall CJ 2013 Alternative strategies in avian scavengers: how subordinate species foil the despotic distribution. Behav. Ecol. Sociobiol. 67, 383–393. (10.1007/s00265-012-1458-5)

[19] Cortés-Avizanda A Jovani R Carrete M Donázar JA 2012 Resource unpredictability promotes species diversity and coexistence in an avian scavenger guild: a field experiment. Ecology 93, 2570–2579. (10.1890/12-0221.1) 23431588

[20] Ogada DL Keesing F Virani MZ 2012 Dropping dead: causes and consequences of vulture population declines worldwide. Ann. NY Acad. Sci. 1249, 57–71. (10.1111/j.1749-6632.2011.06293.x) 22175274

[21] Green RE 2004 Diclofenac poisoning as a cause of vulture population declines across the Indian subcontinent. J. Appl. Ecol. 41, 793–800. (10.1111/j.0021-8901.2004.00954.x)

[22] Ogada D Torchin M Kinnaird M Ezenwa V 2012 Effects of vulture declines on facultative scavengers and potential implications for mammalian disease transmission. Conserv. Biol. 26, 453–460. (10.1111/j.1523-1739.2012.01827.x) 22443166

[23] Sekercioglu CH 2006 Increasing awareness of avian ecological function. Trends Ecol. Evol. 21, 464–471. (10.1016/j.tree.2006.05.007) 16762448

[24] Morand-Ferron J Giraldeau LA 2010 Learning behaviorally stable solutions to producer–scrounger games. Behav. Ecol. 21, 343–348. (10.1093/beheco/arp195)

[25] Barta Z Giraldeau LA 1998 The effect of dominance hierarchy on the use of alternative foraging tactics: a phenotype-limited producing-scrounging game. Behav. Ecol. Sociobiol. 42, 217–223. (10.1007/s002650050433)

[26] Hofbauer J Sigmund K 2003 Evolutionary game dynamics. Bull. Am. Math. Soc. 40, 479–519. (10.1090/S0273-0979-03-00988-1)

[27] Bamford AJ Monadjem A Hardy ICW 2010 Associations of avian facial flushing and skin colouration with agonistic interaction outcomes. Ethology 116, 1163–1170. (10.1111/j.1439-0310.2010.01834.x)

[28] Wilensky U 1999 NetLogo center for connected learning and computer-based modeling. Evanston, IL: Northwestern University See http://ccl.northwestern.edu/netlogo.

[29] Dermody BJ Tanner CJ Jackson AL 2011 The evolutionary pathway to obligate scavenging in *Gyps* vultures. PLoS ONE 6, e24635 (10.1371/journal.pone.0024635) 21931786 PMC3169611

[30] Spiegel O Getz WM Nathan R 2013 Factors influencing foraging search efficiency: why do scarce lappet-faced vultures outperform ubiquitous white-backed vultures? Am. Nat. 181, E102–E115. (10.1086/670009) 23594555

[31] Howland CH Merola S Basarab JR 2004 The allometry and scaling of the size of vertebrate eyes. Vis. Res. 44, 2043–2065. (10.1016/j.visres.2004.03.023) 15149837

[32] Xirouchakis SM Andreou G 2009 Foraging behaviour and flight characteristics of Eurasian griffons *Gyps fulvus* in the island of Crete, Greece. Wildlife Biol. 15, 37–52. (10.2981/07-090)

[33] Ruxton GD Houston DC 2004 Obligate vertebrate scavengers must be large soaring fliers. J. Theor. Biol. 228, 431–436. (10.1016/j.jtbi.2004.02.005) 15135041

[34] Hedenstrom A 1993 Migration by soaring or flapping flight in birds: the relative importance of energy cost and speed. Phil. Trans. R. Soc. Lond. B 342, 353–361. (10.1098/rstb.1993.0164)

[35] Cone CD 1962 Thermal soaring of birds. Am. Sci. 50, 180–209.

[36] Virani MZ Kendall C Njoroge P Thomsett S 2011 Major declines in the abudance of vultures and other scavenging raptors in and around the Masai Mara ecosystem, Kenya. Biol. Conserv. 144, 746–752. (10.1016/j.biocon.2010.10.024)

[37] Kéfi S 2012 More than a meal… integrating non-feeding interactions into food webs. Ecol. Lett. 15, 291–300. (10.1111/j.1461-0248.2011.01732.x) 22313549

[38] Slocombe DS 1993 Implementing ecosystem-based management. BioScience 43, 612–622. (10.2307/1312148)

[39] Layzer JA 2012 The purpose and politics of ecosystem-based management. In Sustainability science: the emerging paradigm and the urban environment (eds Weinstein MP Turner RE), pp. 177–197. New york: Springer.

[40] Ogada DL Keesing F 2010 Decline of raptors over a three-year period in Laikipia, central Kenya. J. Raptor Res. 44, 129–135. (10.3356/JRR-09-49.1)

