# Supplementary material for: Vultures acquire information on carcass location from scavenging eagles
Source: Proc Biol Sci. 2014 Oct 22;281(1793):20141072. doi: 10.1098/rspb.2014.1072 (PMC4173674; doi:10.1098/rspb.2014.1072)
Supplement: Supplementary Information [file rspb20141072supp1.docx]

**Vultures acquire information on carcass location from scavenging eagles –Supplementary Information**

**Details of experimental setup**

The carcasses of partially skinned goat and cow carcasses were set out at dawn (06.15 – 07.20) in an open area of the Mpala Ranch which consists of 20,000 ha of savannah. Carrion size ranged from 20 – 340 kg with a mean of 80 kg and did not have a significant effect on the number of avian scavengers present [1].

**Producer-scrounger model**

**Table S1.** Variables of the game theoretic model system and their definitions.

| **Model Variables** | **Definition** |
| --- | --- |
| *v_s_*  *v_p_*  *r_s_*  *r_p_* | No. of scrounger vultures  No. of producer vultures  No. of scrounger raptors  No. of producer raptors |
| *m_v_*_,v_ | No. of vultures found at a vulture-produced carcass |
| *m*_v,r_ | No. of raptors found at a vulture-produced carcass |
| *m_r_*_,v_ | No. of vultures found at a raptor-produced carcass |
| *m_r_*_,r_ | No. of raptors found at a raptor-produced carcass |
| *π* | Rate of food consumption |
| d (= 1/(1 + *r_p_* + *v_p_*))  *a*  *x*  *α*  *β*  *γ*/(1 + *r_p_* + *r_s_*) | Steady state density of carcasses  Finder’s fee  The competitive ability of vultures compared to that of raptors  Vulture mortality rate  Raptor mortality rate  Food gained by raptors through predation |

**Individual Based Model - differences between vultures and raptors**

Our model reflects biological differences between raptors and vultures by allowing raptors to have an earlier start on a foraging day, to be more widely dispersed in the initialisation of the simulation space and to have an improved detection distance. We scale the relative visual detection abilities of the two groups as a measure of sensitivity analysis. The background for these biological differences is explained here.

Tawny Eagles have an axial length of 26.51 (c.f. White-backed Vultures who measure 20.71) [2]. This gives a measure of visual acuity of 81.5 cycles m^-1^ (again c.f. White-backed Vultures with a measure 57.5 m^-1^) [2, 3]. So this raptor species has better absolute and relative eyesight if we accept these measures, which is further increased by its probable lower flying height [4]. Moreover, raptors can depart earlier in the day owing to their lower wing loading relative to the larger *Gyps* vultures [4]. The social *Gyps* are also more densely aggregated at their roost sites [4] relative to the solitary raptors. Vultures are also known to fly at great altitudes [4]. Vickery *et al.* [5] note that “producer individuals may fly low to increase their probability of detecting a patch when they fly over it”. By flying above the producing raptors the vultures have the potential to notice any raptor that descends to a carcass. Mundy [4] reports a typical altitude of 350m and 300m for the Rüppell's Vulture and White-backed Vulture respectively. Although the flying height of Tawny and Steppe Eagles is unknown, these species occupy a similar ecological niche to the Bateleur Eagle (*Terathopius ecaudatus*) which often feeds on carrion [6]. The Bateleur has been recorded cruising at a height of just 50m above ground [4]. We can justifiably make the assumption that the two raptors that predominate our data fly at a similar altitude while foraging or at least below that of the *Gyps* vultures.

**Table S2.** Parameter values of the individual-based models that were written in NetLogo.

The first row represents the case of equal detection distance between raptors and vultures; the second is where raptors can see twice the distance and so on. Enhanced range relates to instances where a local enhancement effect is at play, i.e. the carcass is already occupied by a bird.

| **No. Raptors** | **No. Vultures** | **Vulture Detection Range** | **Raptor Detection Range** | **Vulture Enhanced Range** | **Raptor Foraging Day (Hrs)** | | **Vulture Foraging Day (Hrs)** | |
| --- | --- | --- | --- | --- | --- | --- | --- | --- |
| 0 - 10 | 90 | 1km | 1km | 4km | 7 | 5 | |  |
| 0 - 10 | 90 | 1km | 2km | 4km | 7 | 5 | |  |
| 0 - 10 | 90 | 1km | 3km | 4km | 7 | 5 | |  |
| 0 - 10 | 90 | 1km | 4km | 4km | 7 | 5 | |  |

**References**

1. Ogada D, Torchin M, Kinnaird M, Ezenwa V. 2012 Effects of Vulture Declines on Facultative Scavengers and Potential Implications for Mammalian Disease Transmission. *Conserv Biol*. **26**, 453-460.

2. Howland CH, Merola S, Basarab JR. 2004 The allometry and scaling of the size of vertebrate eyes. *Vision Res.* **44,** 2043-2065.

3. Spiegel O, Getz WM, Nathan R. 2013 Factors Influencing Foraging Search Efficiency: Why Do Scarce Lappet-Faced Vultures Outperform Ubiquitous White-Backed Vultures? *Am Nat.* **181, E**102-E115.

4. Mundy PJ, Butchart D, Ledger J, Piper S. 1992 *The vultures of Africa*: Academic Press London.

5. Vickery WL, Giraldeau LA, Templeton JJ, Kramer DL, Chapman CA. 1991 Producers, scroungers, and group foraging. *Am Nat*.**137**, 847-863.

6. Steyn P. 1980 Breeding and food of the bateleur in Zimbabwe (Rhodesia). *Ostrich*.**51**, 168-178.
